# Gene Mutation-Negative Malignant Melanoma in a Prepubertal Patient: A Clinical and Molecular Case Report

**DOI:** 10.3390/genes16080937

**Published:** 2025-08-06

**Authors:** Adrian Guźniczak, Patrycja Sosnowska-Sienkiewicz, Jarosław Szydłowski, Paweł Kurzawa, Danuta Januszkiewicz-Lewandowska

**Affiliations:** 1 Faculty of Medicine, Poznan University of Medical Sciences, Fredry 10 Street, 61-701 Poznan, Poland; guzniczak.adrian@gmail.com; 2 Department of Pediatric Surgery, Medical University of Warsaw, Żwirki i Wigury 63A Street, 02-091 Warsaw, Poland; 3 Department of Pediatric Otolaryngology, Poznan University of Medical Sciences, 27/33 Szpitalna Street, 60-572 Poznan, Poland; szydlowski@ump.edu.pl; 4 Department of Clinical Pathology, Poznan University of Medical Sciences, Przybyszewskiego 49 Street, 60-355 Poznan, Poland; paul.kurzawa@yahoo.com; 5 Department of Paediatric Oncology, Hematology and Transplantology, Poznan University of Medical Sciences, 27/33 Szpitalna Street, 60-572 Poznan, Poland; danuta.januszkiewicz@ump.edu.pl

**Keywords:** melanoma, child, molecular testing

## Abstract

Conventional melanoma is exceedingly rare in the pediatric population, particularly among prepubescent children, and its diagnosis and management necessitate a multidisciplinary approach. The objective of this present report is to delineate the diagnostic pathway and therapeutic management of a 4-year-old girl with conventional melanoma, with particular focus on the molecular context. A pigmented lesion located on the auricle was surgically excised, and subsequent histopathological and immunohistochemical analyses confirmed the diagnosis of malignant melanoma (pT3b). Radiologic investigations revealed no evidence of metastatic disease, and comprehensive genetic testing utilizing next-generation sequencing (NGS) identified no pathogenic variants in the germline genes examined, nor in the *BRAF*, *NRAS*, *KRAS*, and *TP53* genes within the excised lesion. The patient remains in good general health. This case report adds to the limited body of literature on melanoma in pediatric patients and underscores the importance of thorough diagnostic evaluation in this age group.

## 1. Introduction

Melanoma is a malignancy originating from melanocytes—the pigment-producing cells that synthesize melanin. These cells are found primarily in the skin but also in other organs [1]. Cutaneous melanoma is potentially the most dangerous form of skin cancer and accounts for 90% of deaths due to skin cancer [2]. Melanoma is the third most common cancer among young adults aged 20–39 years in the United States [3]. In contrast, it is rare in children and adolescents. Among children aged 14 years or younger, melanoma accounts for about 1% of all cancers, with an estimated incidence of 1.6 per million individuals [4]. In adolescents aged 15–19 years, the incidence rises to 8.2 per million, representing 3% of all cancers in this age group [4]. As in adults, most reported cases in children and adolescents are cutaneous melanoma (96.3) and often arise de novo (70.9%) [5,6]. Melanomas in children, particularly before puberty, more often present as amelanotic, nodular lesions of greater thickness at the time of diagnosis compared to adult melanomas. A significant proportion of cases (60% in children < 10 years and 40% in those aged 11–19 years) do not meet the classic ABCDE criteria, which include asymmetry, border irregularity, color variation, diameter > 6 mm, and evolution. In response, pediatric ABCD criteria have been proposed, including: amelanotic, bleeding/bump, uniform color, de novo, and any change [7].

In 2018, the World Health Organization classified nine melanoma pathways based on cumulative sun damage (CSD) and associated genetic mutations [8,9]. In children, melanomas represent a heterogeneous group that can be divided into three categories: spitzoid melanoma, melanoma arising in congenital melanocytic nevi, and conventional melanoma—histologically indistinguishable from adult cases [10,11,12]. The frequency of each of these three subtypes varies with age, with spitzoid melanoma being more common in those under 12 years, while melanoma arising in congenital nevi and conventional melanoma are more frequent in adolescents [10,11]. Superficial spreading melanoma (SSM) and nodular melanoma are the most common histologic types among conventional melanoma cases, with nodular melanoma being more frequent [10,11]. Studies indicate that the SSM subtype most often harbors *BRAF* mutations (64.3%), and many also carry *TERT* promoter and *CDKN2A* mutations. Mutations in *CCND1* (6%) are less frequent, while *PTEN* and *TP53* mutations are observed in more advanced tumors [13]. In contrast, nodular melanoma shows *BRAF* mutations in 36.4–47% of cases but is more often associated with *NRAS* mutations (27%) and alterations in *PD-L1* regulation [13].

Surgical resection remains the standard of care in pediatric melanoma, with prognosis strongly dependent on disease stage [14,15].

Melanoma is a multifactorial skin cancer arising from the interaction between environmental exposure and genetic susceptibility. In children, major risk factors include pediatric-specific conditions such as giant CMN, xeroderma pigmentosum, immunodeficiency, and vitiligo [16]. Ultraviolet radiation (UVR) is the most significant known etiologic factor associated with melanoma [17]. The risk associated with UVR depends on latitude, cumulative exposure, pattern of exposure, and history of sunburns, and it correlates with certain phenotypic features such as fair skin, red/blond hair, light eyes, freckles, sunburn susceptibility, presence of numerous melanocytic nevi, and dysplastic nevus syndrome [17,18,19]. It is unclear why melanoma rarely occurs at a young age, and its biological underpinnings are not fully understood, highlighting the role of inherited germline variants that predispose to melanoma [20].

The literature on the molecular pathogenesis of pediatric melanoma is limited, and reports of patients under 10 years of age are anecdotal. Therefore, the aim of this article is to provide a detailed description of the diagnostic process, including genetic testing, and therapeutic management of a 4-year-old girl with conventional melanoma, with the goal of raising awareness about the possibility of melanoma occurring in this age group as well.

## 2. Case Description

**Figure 1:**
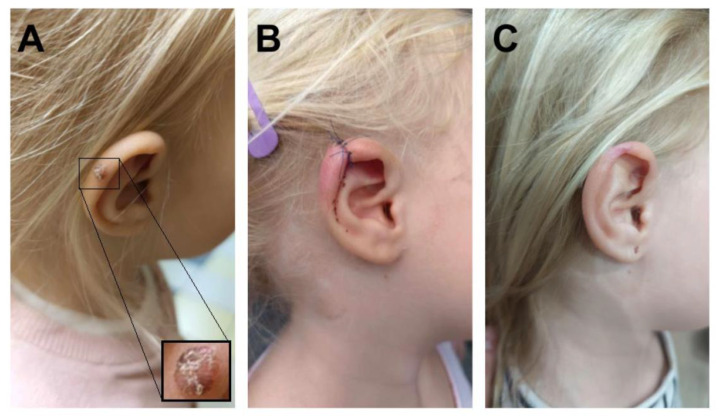
(**A**) Melanocytic lesion on the patient’s left ear. (**B**) Reconstruction by primary closure. (**C**) Post-healing status.

A 4-year-old Caucasian girl presented in 2023 to a surgeon due to a pigmented lesion on the right ear that had appeared in 2022. The parents were initially advised to observe the lesion; however, after two months, they noticed enlargement of the lesion and changes in its color and surface texture. The lesion did not cause pain or itching. During a subsequent surgical visit, it was decided to excise the brown nodule with ulceration (on the surface and border) on the right ear, measuring up to 0.4 cm at its greatest dimension (Figure 1). Histological examination revealed a diagnosis of malignant melanoma. The parents were then referred for an oncological consultation. At the time of the oncology visit, physical examination revealed only a small (0.5 cm), well-healed scar on the auricle. The child was the only child of healthy parents. She was developing normally and had been vaccinated according to the Polish national immunization schedule. Both the parents and the child had very fair skin and blue eyes. Family history revealed malignancies in relatives: breast cancer in the maternal great-grandmother, kidney cancer in the maternal great-grandfather, and pancreatic cancer in the paternal grandmother.

**Figure 2:**
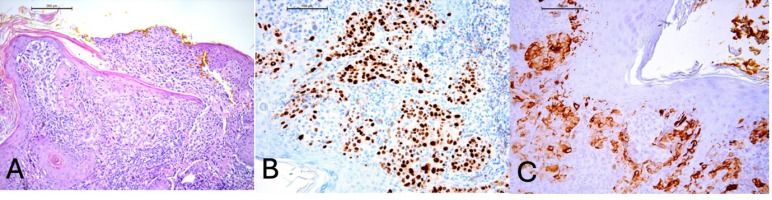
(**A**) Melanocytes infiltrating and disrupting the epidermis, H&E, 100× magnification. (**B**) Positive staining for PRAME (preferentially expressed antigen in melanoma) in tumor cells, 200× magnification. (**C**) Positive staining for HMB45 (HMB45 is a monoclonal antibody directed against the gp100 protein, which is a component of melanosomes) in tumor cells, 200× magnification.

Due to the unique diagnosis of melanoma at such a young age, histopathological examination was reviewed, including the assessment of resection margins. The histopathological findings demonstrated an atypical melanocytic lesion with features of severe dysplasia. Nests of atypical melanocytes were present in the epidermis and dermis, along with ulceration and pagetoid spread of melanocytes within the epidermis (Figure 2A). No signs of maturation were observed. The minimum lateral margin of uninvolved tissue was 0 mm, and the minimum deep margin was 1.4 mm. The invasion depth was 2.4 mm. Atypical melanocytes were present in the reticular dermis (Clark level 4) with an elevated Ki67 proliferation index, focally up to 40%. Immunohistochemical (IHC) staining revealed SOX-10 (+), PRAME (+), HMB45 (+ in single cells), p16 (patchy +), and elevated Ki67 (focally up to 40%) (Figure 2B,C). A second macroscopic report (printout no. 81129) additionally noted Melan (+), HMB45 (+), S-100P, and p16 (+) in microclusters of the proliferative tissue, partly nuclear, partly artifactual. The histological findings combined with the immunohistochemical results raised suspicion of invasive melanoma (pT3b). Therefore, a second surgical procedure was performed, involving an arched excision of the skin lesion along with a portion of the right auricular cartilage, achieving a 4 mm lateral margin and 3 mm deep margin, which was confirmed histopathologically (Figure 1B). Given the patient’s history, additional imaging studies (ultrasound and CT) were performed to exclude metastatic lesions in the chest, abdomen, head, and locoregional lymph nodes. No abnormalities were detected in these examinations.

Because of the patient’s young age, genetic testing (both constitutional DNA and tumor tissue) was performed to assess for mutations in genes associated with melanoma development. Germline testing was performed using the Blueprint Genetics Hereditary Pediatric Cancer Panel Plus (version 4, 19 October 2019). The test was performed in a certified laboratory using next-generation sequencing (NGS) on an Illumina platform. The panel includes full sequence and copy number variation (CNV) analysis of 71 genes (Table 1) relevant to cancer predisposition in children and adolescents. It targets all protein coding exons, exon–intron boundaries (± 20 base pairs), and selected non-coding deep intronic variants known to be clinically relevant. The median sequencing depth was 127×, with 99.82% of target bases covered at >20×. The test allows for detection of single nucleotide variants (SNVs), small insertions and deletions (INDELs), and exon-level CNVs. In addition to the NGS-based germline analysis, targeted testing for the CDKN2A gene was performed using a quantitative PCR (qPCR) assay. The test was conducted in a certified diagnostic laboratory. The estimated analytical sensitivity of the qPCR method was approximately 99%. Internal quality control was ensured by analyzing each patient sample alongside validated positive and negative controls, confirming the specificity and accuracy of the assay. Somatic mutation analysis in tumor tissue was also carried out, targeting BRAF, TP53, KRAS, and NRAS genes. None of these tests revealed pathogenic variants in the genes analyzed.

The patient remains under regular oncological follow-up every three months, including ultrasound assessments of the abdomen and lymph nodes. The parents adhere to recommendations regarding sun protection measures. At the time of preparation of this manuscript, the child was in good general condition, with no evidence of recurrence at the excision site (Figure 1C)**.**

## 3. Discussion

In this case report of conventional melanoma in a very young child, we integrated clinical history, histopathological findings, and molecular genetic analysis, offering valuable insight into pediatric melanoma. Given the rarity of conventional melanoma in prepubescent patients and the limited use of comprehensive genetic testing, this multifaceted diagnostic approach is uncommon in the current literature. A notable limitation of our study is its single-case design, which precludes broad generalizations regarding pediatric melanoma at this age. Future investigations should include additional genetic analyses—particularly of rare low- and moderate-penetrance variants—to strengthen understanding in this demographic.

Interesting studies addressing related topics include, among others, a study by Charles Lu et al., which performed genome sequencing of 15 pediatric cases, revealed a high UV-related mutational burden, numerous C>T mutations at dipyrimidine sites, activating mutations in *BRAF* and *TERT* promoter genes, and frequent inactivation of tumor suppressor genes *CDKN2A* and *PTEN* following *BRAF* activation. In most cases patients carried one or two of the four most common germline *MC1R* variants [12]. Similarly, Pappo et al. evaluated 17 pediatric patients aged 10–18 years with conventional melanoma. Eleven harbored the BRAFV600E mutation and seven TERT promoter mutations [23]. Additional genetic aberrations identified among the cohort included mutations in *NRAS*, *ATM*, *CTNNB1*, *MAP2K1*, and *JAK3*; deletions of *CDKN2A*, *CDKN2B*, and *PTEN*; *ALK* gene rearrangements; and a *ZNF38–MARS* fusion [23,24]. In one patient who experienced disease relapse, a TSC2 mutation was also noted, together with amplification and copy number gains in multiple genes including *CDK3*, *KIT*, *MYC*, *MDM2*, and *RAC1*. The youngest patient included in the study was a 10-year-old girl diagnosed with Stage I melanoma located on the trunk; only a BRAF mutation was detected in her tumor. However, comprehensive genomic profiling—such as whole-genome, whole-exome, or transcriptome sequencing—and targeted panel sequencing, performed in other selected patients from the cohort, were not conducted in her case. In contrast, our patient, diagnosed at 4 years old, underwent extensive genetic testing, including a comprehensive germline mutation panel covering 72 genes commonly associated with hereditary pediatric cancers. Despite this broad assessment, no pathogenic variants were identified in either germline or somatic testing. This highlights not only the rarity of conventional melanoma at such a young age but also the added diagnostic value of integrating advanced molecular profiling, which is not routinely performed in all pediatric cases reported in the literature.

Church et al. also demonstrated a high UV-induced mutational burden in conventional melanoma across both children and adults [25]. In our patient, analyses of UV-induced mutation burden were not performed. It is important to note that mutational signature analysis is not currently recommended in routine melanoma diagnostics according to NCCN and ESMO guidelines, and its use is primarily limited to translational research [26,27]. However, analyzing UV-induced mutations appears valuable for a deeper understanding of cutaneous melanoma development, especially in patients diagnosed before puberty. In our patient, the history was negative for past sunburns, and the patient’s mother reported that broad-spectrum sunscreen (SPF 50) had been applied regularly over the child’s entire body. In melanomas of young adults, non–UV-associated mutational processes are observed, including signs of APOBEC enzyme activity and defects in mismatch repair pathways [28]. Furthermore, although melanoma commonly arises after years of cumulative UV damage, its occurrence in childhood—though rare—suggests potential underlying genetic predispositions.

In adults, melanoma susceptibility is thought to involve polygenic inheritance of high-, moderate-, and low-risk alleles, but genetic information in pediatric patients remains sparse [29]. Low-risk gene variants are common in the population but have low penetrance, in contrast to high-risk genes, which are rare but highly penetrant for cutaneous melanoma development. Rare, high-penetrance, melanoma-predisposing gene variants (*CDKN2A*, *CDK4*, *BAP1*, *POT1*, *ACD*, *TERF2IP*, and *TERT*) confer very high susceptibility to melanoma, resembling a monogenic disorder with autosomal dominant inheritance. Rare, moderate-penetrance variants (*MC1R*, *MITF*, and *SLC45A2*) and low-penetrance variants (*TYR*, *OCA2*, *ASIP*, *PL2G6*, *FTO*, *PARP1*, *ATM*, *CDKAL1*, *CCND1*, and *CYP1B1*) alone do not cause melanoma but contribute to polygenic susceptibility and can significantly increase risk [21]. High-risk genes in adults, such as CDKN2A and CDK4, seem to have little impact in sporadic pediatric melanoma. However, in familial or multiple primary pediatric melanomas, CDKN2A mutations are common [30,31,32].

In the largest study to date on melanoma in patients under 21 years of age, Pellegrini et al. (*n* = 123) identified *CDKN2A* mutations in 2% of sporadic cases. No mutations were found in *CDK4* or *POT1*. Most subjects (67%) carried *MC1R* variants, and the *MITF E318K* mutation was present in 2% of patients. Patients with sporadic melanoma differed significantly from familial/multiple cases by younger age at diagnosis, less frequent red hair, fewer nevi, and lower frequency of pathogenic *CDKN2A* and *MC1R R160W* variants. The *MC1R V92M* variant was more frequent in children than adolescents. The mean age at diagnosis was 14.6 years (range: 4–21); no individual patient data are available [32]. In our patient, no mutations were detected in the tested genes, including *CDKN2A* or *CDK4*, consistent with the latest scientific reports on conventional melanoma in the youngest patients. However, the molecular panel used did not cover some rare moderate- and low-penetrance genes, which was due to the use of a targeted diagnostic strategy focused on genes with the highest clinical relevance. The selected Hereditary Pediatric Cancer Panel Plus allowed for comprehensive assessment of cancer predisposition, balancing diagnostic breadth, clinical utility, and economic feasibility. Given the family history of breast and pancreatic cancers in relatives, the absence of BRCA2 in the selected panel may be considered a limitation. Although BRCA2 is not among the genes typically implicated in pediatric melanoma, it is a well-established predisposition gene for both breast and pancreatic cancer, and its inclusion could have provided additional clinical insight in this case [33]. In addition, somatic profiling in our study included analysis of several key genes (BRAF, NRAS, KRAS, TP53). Current ESMO guidelines recommend testing for BRAF mutations in patients eligible for systemic treatment, which is primarily for cases in stages III–IV or in situations where adjuvant treatment or participation in clinical trials is being considered [27]. However, the scope of our analysis did not include the full somatic panel, which could be particularly useful in atypical cases such as melanoma in a child. Expanding the study to include other relevant alterations—such as TERT promoter mutations, CDKN2A deletions, PTEN loss, MITF amplification, or MC1R variants—could add diagnostic value, especially given the patient’s young age and lack of pathogenic germline alterations.

Particular attention should be paid to *MC1R* or *MITF E318K* variants, which may influence melanoma risk in young children. *MITF* regulates melanocyte development, and the p.E318K variant is associated with numerous nevi, fair complexion, blue eyes, and increased risk of melanoma and renal cell carcinoma [34,35]. *MC1R* is highly polymorphic in populations of European descent [36], and its variants are classified as “R” or “r” alleles depending on their association with the red hair phenotype, which includes fair skin, red hair, freckles, UV sensitivity, and poor tanning ability [37,38]. Carriers of any *MC1R* variant among Caucasians have been shown to have a 66% higher risk of melanoma compared to wild-type carriers, regardless of phenotype [37]. Germline MC1R variants are not significantly associated with the frequency of somatic mutations in BRAF or NRAS, and their influence on melanoma risk in tumors with these mutations is at most moderate. Furthermore, certain expected phenotypic and tumor-specific characteristics have been observed, including higher prevalence of red hair and Fitzpatrick skin types I–II, greater nevus counts, and more frequent superficial spreading melanomas in familial cases. Our patient presented with Fitzpatrick type II phenotype, which is characterized by fair complexion, blonde hair, blue eyes, a tendency to burn easily, and minimal, light tanning. The patient’s parents were Caucasian, also characterized by fair complexion, light hair, and blue eyes, which may suggest the presence of moderate- and low-penetrance susceptibility alleles associated with a melanoma-promoting phenotype. Fair, sunburn-prone skin is the most observed phenotypic risk factor, whereas the strongest exogenous risk factor for cutaneous melanoma remains ultraviolet (UV) exposure, particularly intermittent exposure [39,40].

The mainstay of treatment is surgical resection. All suspicious skin lesions should undergo punch, incisional, or excisional biopsy. Sentinel lymph node biopsy (SLNB) is indicated for all T1b or higher lesions, as well as those 0.8–1 mm thick with worrisome features (mitoses > 2/mm^2^, ulceration, or lymphovascular invasion) [14]. As in adults, melanoma-specific prognosis is favorable in early stages but poor in patients with metastases [15]. Five-year relative survival is highest in children and decreases with increasing age. Studies have shown that 5-year survival is 96 ± 1.2% for patients ≤ 14 years, 95.5 ± 0.2% for those 15–19 years, and 91.8 ± 0.1% in the 20–39 age group [3]. Survival also depends on disease stage: 5-year survival in children ≤ 17 years is 99% for in situ melanoma, 98% for stage I, 94.6% for stage II, 91.4% for stage III, and 34.4% for stage IV [41]. For comparison, adult 5-year survival is lower for stage III (75%) but similar in other stages [4].

In our patient, sentinel lymph node biopsy (SLNB) was not performed. The decision was made by a multidisciplinary team in agreement with the patient’s parents, based on a risk–benefit analysis that considered the child’s very young age and the absence of clinical signs of regional metastases.

Giant congenital melanocytic nevus (GCMN) is a lesion that can give rise to malignant melanoma. Surrenti et al. reported a case of a 5-year-old child with a giant congenital melanocytic nevus who developed malignant melanoma characterized by rapid progression, metastatic spread, and ultimately, a fatal outcome. This case underscores the critical importance of thorough and regular full-body skin examinations during follow-up in patients with GCMN, as well as the potential value of early prophylactic surgical intervention [42]. In conclusion, although rare, they pose significant oncological and neurological risks, including malignant transformation and neurocutaneous melanocytosis [43]. Mahajan et al. and Scalvenzi et al. likewise underscore comparable conclusions in their respective studies [44,45]. Although our patient did not present with GCMN, this topic is important to mention in the context of melanoma development.

## 4. Conclusions

Pediatric melanoma remains a rare but clinically and genetically heterogeneous condition. While UV-induced damage plays a significant role in many cases, younger children may develop melanoma despite the absence of typical environmental risk factors, highlighting the importance of genetic predisposition. Regular follow-up is crucial- particularly in high-risk patients with congenital nevi- along with early recognition and, in selected cases, the potential benefits of prompt surgical intervention. Despite advances in understanding molecular mechanisms, standardized diagnostic criteria and treatment guidelines for pediatric melanoma are still lacking, underscoring the need for an individualized, multidisciplinary approach to each patient.

## Figures and Tables

**Table 1:** List of genes analyzed in the Hereditary Pediatric Cancer Panel Plus. Genes marked with one (*) or two (**) asterisks have a potential association with germline predisposition to melanoma (see legend below).

*BUB1B*	*BRAF*	*BMPR1A*	*BLM*	***BAP1*** *	*AXIN2*	*APC*	*ALK*
*EZH2*	*EPCAM*	*DIS3L2*	*DICER1*	*CEBPA*	*CDKN1C*	*CDC73*	*CBL*
*MAP2K2*	*MAP2K1*	*LZTR1*	*KRAS*	*HRAS*	*GPC3*	*GATA2*	*FH*
*NF2*	***NF1*** **	*NBN*	***MSH6*** **	***MSH2*** **	***MLH1*** **	*MEN1*	*MAX*
*PRKAR1A*	*PRF1*	***PMS2*** **	*PHOX2B*	*PAX5*	*NSUN2*	*NSD1*	*NRAS*
*RET*	*REST*	*RECQL4*	*RASA2*	*RAF1*	*PTPN11*	***PTEN*** *	***PTCH1*** **
*SDHD*	*SDHC*	*SDHB*	*SDHAF2*	*SDHA*	*RUNX1*	*RRAS*	*RIT1*
***SUFU*** **	***STK11*** **	*SOS2*	*SOS1*	*SMARCB1*	*SMARCA4*	*SMAD4*	*SHOC2*
	*WT1*	***WRN*** **	*VHL*	*TSC2*	*TSC1*	***TP53*** *	*TMEM127*

* Gene identified as a germline risk factor for melanoma [21,22]. ** Gene associated with a hereditary syndrome that increases melanoma risk [22].

## Data Availability

Due to restrictions, the data supporting this publication are available upon reasonable request from the corresponding author.

## Abbreviations

The following abbreviations are used in this manuscript:

NGS	next-generation sequencing
INDELs	small insertions and deletions
CNV	copy number variation
SNVs	single nucleotide variants
CSD	cumulative sun damage
SSM	superficial spreading melanoma
SLNB	sentinel lymph node biopsy
UVR	ultraviolet radiation
CT	computed tomography
DNA	deoxyribonucleic acid
PRAME	preferentially expressed antigen in melanoma
GCMN	giant congenital melanocytic nevus
